# QTL mapping for the flag leaf-related traits using RILs derived from *Trititrigia* germplasm line SN304 and wheat cultivar Yannong15 in multiple environments

**DOI:** 10.1186/s12870-024-04993-x

**Published:** 2024-04-18

**Authors:** Xia Zhang, Piyi Xing, Caicai Lin, Honggang Wang, Yinguang Bao, Xingfeng Li

**Affiliations:** 1 Shandong Provincial Key Laboratory of Biophysics, Institute of Biophysics, Dezhou University, Dezhou, Shandong 253023 China; 2 National Key Laboratory of Wheat Improvement, Shandong Agricultural University, Tai’an, Shandong 271018 China; 3 Tai’an Subcenter of the National Wheat Improvement Center, Agronomy College, Shandong Agricultural University, Tai’an, Shandong 271018 China

**Keywords:** Wheat (*Triticum aestivum* L.), *Th. intermedium*, Flag leaf traits, QTLs, Candidate genes

## Abstract

**Background:**

Developing and enriching genetic resources plays important role in the crop improvement. The flag leaf affects plant architecture and contributes to the grain yield of wheat (*Triticum aestivum* L.). The genetic improvement of flag leaf traits faces problems such as a limited genetic basis. Among the various genetic resources of wheat, *Thinopyrum intermedium* has been utilized as a valuable resource in genetic improvement due to its disease resistance, large spikes, large leaves, and multiple flowers. In this study, a recombinant inbred line (RIL) population was derived from common wheat Yannong15 and wheat-*Th. intermedium* introgression line SN304 was used to identify the quantitative trait loci (QTL) for flag leaf-related traits.

**Results:**

QTL mapping was performed for flag leaf length (FLL), flag leaf width (FLW) and flag leaf area (FLA). A total of 77 QTLs were detected, and among these, 51 QTLs with positive alleles were contributed by SN304. Fourteen major QTLs for flag leaf traits were detected on chromosomes 2B, 3B, 4B, and 2D. Additionally, 28 QTLs and 8 QTLs for flag leaf-related traits were detected in low-phosphorus and drought environments, respectively. Based on major QTLs of positive alleles from SN304, we identified a pair of double-ended anchor primers mapped on chromosome 2B and amplified a specific band of *Th. intermedium* in SN304. Moreover, there was a major colocated QTL on chromosome 2B, called *QFll/Flw/Fla-2B*, which was delimited to a physical interval of approximately 2.9 Mb and contained 20 candidate genes. Through gene sequence and expression analysis, four candidate genes associated with flag leaf formation and growth in the QTL interval were identified.

**Conclusion:**

These results promote the fine mapping of *QFll/Flw/Fla-2B*, which have pleiotropic effects, and will facilitate the identification of candidate genes for flag leaf-related traits. Additionally, this work provides a theoretical basis for the application of *Th. intermedium* in wheat breeding.

**Supplementary Information:**

The online version contains supplementary material available at 10.1186/s12870-024-04993-x.

## Introduction

Wheat (*Triticum aestivum* L.) is one of the most important cereal crop species worldwide. Ensuring high wheat production is necessary to meet the food demand of a growing human population [1]. *Thinopyrum intermedium* is an important wild relative of wheat that possesses excellent characteristics, such as disease and insect resistance, as well as stress resistance [2]. Furthermore, some potentially essential disease resistance genes from *Th. intermedium* have been introduced into common wheat [3–7]. Moreover, *Th. intermedium* exhibits valuable agronomic traits, including large spikes, diverse flag leaf traits, and multiple flowers, which provide abundant phenotypic variation for wheat breeding [2, 7].

Flag leaf-related traits are associated with many important agronomic traits related to wheat growth and development, such as plant height, kernel number per spike, yield, and stress responses [8, 9]. Several studies have shown that flag leaf size has a positive effect on thousand-kernel weight and kernel number per spike [10–14]. Flag leaves are the main organ for photosynthesis, and they play a crucial role in increasing yield and facilitating photosynthesis [15–17]. Additionally, some researchers have shown that the flag leaf supplies approximately 50% of the total photosynthetic activity and approximately 41-43% of the carbohydrates required for grain filling [18]. Consequently, breeding wheat with the best flag leaf size has been proposed as a viable approach for increasing grain yields.

With the availability of molecular markers and genetic maps, numerous quantitative trait loci (QTLs) related to flag leaf-related traits have been discovered in rice, barley and wheat [19–22]. In rice, Chen et al. reported a flag width QTL, *qFLW4*, which contains a narrow *NAL1* gene with a 74.8 kb interval [23]. Tang et al. discovered 14 QTLs for FLL and 9 QTLs for FLW in the CSSL population, with *qFLW7.2* mapping to a 37 kb interval for FLW [24]. Wang et al. discovered 64 QTLs for flag leaf-related variables using two recombinant inbred line (RIL) populations and identified five candidate genes controlling flag leaf width [25]. In barley, researchers discovered 38 QTLs for flag leaf on chromosomes 1 H, 2 H, 3 H, 4 H, 6 H, and 7 H; two QTLs on chromosomes 5 H and 7 H; and two QTLs on chromosome 5 H, as well as a new major QTL for flag leaf thickness on chromosome 3 H with a logarithm of odds (LOD) value of 18.4 that explained 32% of the phenotypic variation [26–28].

In wheat, QTL analysis of flag leaf-related traits has been reported on 21 chromosomes. In different environments, researchers have discovered seven QTLs for FLL, 11 QTLs for FLW, and 13 QTLs for FLA using an RIL population [29]. Four QTLs for FLL, two for FLW, and four for FLA were discovered in at least two environments distributed on chromosomes 1B, 2B, 3 A, 3D, 4B, 5 A, 6B, 7B, and 7D, and individual QTLs accounted for 4.3-68.52% of the phenotypic variation in multiple environments [30]. Thirty-one QTLs for flag leaves were found in four environments, with two QTLs for FLL on chromosomes 3B and 4 A (*QFll-3B* and *QFll-4 A*) and one for FLW on chromosome 2 A (*QFlw-2 A*) as a crucial stable QTL that affects yield-related traits [31]. QTL identification and validation for flag leaf traits in seven different parental populations in 11 environments using the newly developed KASP markers revealed eight major QTLs explaining 5.73-54.38% of the phenotypic variation [32]. In the case of FLW, some studies reported new stable QTLs, namely, *QFlw-5B, TaFLW1*, and *QFLW-6 A*, which lay the foundation for further fine-mapping and cloning of the gene [19, 33, 34].

These QTLs and genes can change the physiological function of plants by regulating leaf morphology, and they have a significant impact on the coordination of light energy use and the “sink-source” connection.

In this work, we established a recombinant inbred line (RIL) population derived from common wheat YN15 and wheat-*Th. intermedium* introgression line SN304. The objectives of this study were to (i) measure the characteristics of flag leaves in RIL populations in multiple environments; (ii) identify major and stable QTLs for FLL, FLW, and FLA using a SLAF-seq genetic map; (iii) use double-end anchored primers to explore the relationships between these major QTLs and *Th. intermedium* chromosome segments; and (iv) screened candidate genes related to flag leaf development. As a result, we may have a greater understanding of the genetic basis for wheat flag leaf traits, and *Th. intermedium* may offer beneficial tools for breeding and increasing yield potential in wheat.

## Results

### Phenotypic performance and correlation analysis

Significant differences were observed between parents from the RIL population. The values of the flag leaf traits of SN304 were greater than those of YN15 in all environments, and the average RILs were between those of the parents in most environments (Table 1).

In low-phosphorus environments (E2, E4, E7, and E10), the average RILs for flag leaf-related traits were significantly different from those in normal phosphorus environments (E1, E3, E6, and E9). From 2016 to 2019, FLL, FLW, and FLA tended to decrease in low-phosphorus environments, indicating that low-phosphorus environments have a great impact on flag leaves. Under drought conditions (E5 and E8), the flag leaf traits also tended to decrease in the drought-affected environment, especially in FLA, compared with those in the irrigated environment (E3 and E6), showing that the impact on the FLA was more significant. Moreover, the broad-sense heritability of flag leaf length, flag leaf width, and flag leaf area reached 0.71, 0.87, and 0.83, respectively (Table 1). Furthermore, the skewness and kurtosis for FLL, FLW, and FLA showed a pattern of continuous distribution (Additional file 1). The mean squares and F values showed that the flag leaf traits varied greatly among the different environments.

**Table 1:** Phenotype variation for the flag leaf-related traits in different environments

Traits Environment		Parents RILs population										
		YN15	SN304	Mean	SD	Range	*C.V* (%)	Skewness	Kurtosis	Mean squares	F-value	*H* ^*2*^
FLL	2016TAN (E1)	21.74	27.18	25.66	3.30	17.95–36.62	12.85	0.43	0.25	15.81	2.51	0.92
	2016TAL (E2)	18.42	25.58	23.87	3.12	16.15–32.53	13.05	0.18	-0.16	14.26	2.26	
	2017TAN (E3)	21.95	33.38	27.22	3.14	18.74–35.66	11.53	0.30	-0.16	44.83	17.88	
	2017TAL (E4)	20.79	26.69	22.06	2.36	16.61–28.91	10.67	0.09	-0.30	13.38	5.33	
	2017ZB (E5)	18.35	24.93	19.35	1.71	15.36–24.88	8.84	0.33	0.12	23.65	14.38	
	2018TAN (E6)	17.98	23.44	21.28	2.55	15.71–32.95	11.99	0.76	1.55	42.62	17.48	
	2018TAL (E7)	16.38	20.97	16.50	2.11	11.48–22.79	12.81	0.10	-0.06	8.19	3.36	
	2018ZB (E8)	12.26	13.15	12.97	1.44	9.38–16.93	11.13	0.36	0.16	3.42	2.08	
	2019TAN (E9)	19.90	27.02	22.77	2.74	16.8-32.01	12.01	0.50	0.16	24.61	12.56	
	2019TAL(E10)	14.41	20.50	17.37	2.15	11.62–24.5	12.38	0.45	0.16	10.14	5.17	
	BLUP	19.46	24.66	21.19	3.09	16.84–26.5	14.58	0.33	-0.22	44.26	14.74	
FLW	2016TAN (E1)	2.22	2.57	2.12	0.19	1.64–2.60	8.72	0.02	-0.35	0.06	3.59	0.94
	2016TAL (E2)	1.79	2.44	2.05	0.19	1.52–2.70	9.29	0.21	0.29	0.0545	3.42	
	2017TAN (E3)	2.07	2.64	2.33	0.21	1.87–3.38	8.83	0.71	2.18	0.17	9.77	
	2017TAL (E4)	2.05	2.46	2.08	0.17	1.63–2.56	8.09	0.08	-0.15	0.08	4.45	
	2017ZB (E5)	1.91	2.28	1.89	0.21	1.58–3.99	10.86	0.60	0.49	0.12	5.43	
	2018TAN (E6)	1.77	2.19	1.94	0.20	1.47–2.88	10.08	1.06	2.87	0.15	13.36	
	2018TAL (E7)	1.52	2.13	1.68	0.15	1.31–2.14	9.23	0.40	0.15	0.05	4.69	
	2018ZB (E8)	1.45	1.54	1.49	0.12	1.20–1.85	8.26	0.32	0.06	0.04	1.66	
	2019TAN (E9)	2.04	2.42	2.09	0.18	1.56–2.57	8.65	0.13	-0.32	0.09	12.66	
	2019TAL(E10)	1.67	2.13	1.81	0.15	1.44–2.21	8.39	0.27	-0.23	0.05	6.93	
	BLUP	1.86	2.26	1.98	0.02	1.68–2.30	1.01	0.15	-0.31	0.16	11.58	
FLA	2016TAN (E1)	36.18	48.85	38.47	6.35	25.67–53.66	16.51	0.16	-0.74	62.64	2.52	0.92
	2016TAL (E2)	18.74	38.83	35.04	6.37	18.39–51.96	18.17	0.19	-0.07	57.55	2.31	
	2017TAN (E3)	33.93	64.99	46.84	7.55	29.91–70.91	16.12	0.35	-0.19	261.23	17.23	
	2017TAL (E4)	32.44	48.24	34.45	5.51	21.95–52.10	15.99	0.14	-0.14	74.15	4.89	
	2017ZB (E5)	26.56	42.92	27.60	4.07	19.23–43.43	14.76	0.72	0.77	98.31	12.23	
	2018TAN (E6)	23.74	38.27	30.04	5.57	18.15–55.17	18.56	0.92	1.96	153.27	15.49	
	2018TAL (E7)	18.75	32.69	20.30	4.01	11.59–33.38	19.76	0.53	0.20	33.96	3.43	
	2018ZB (E8)	13.29	15.63	14.70	2.60	9.19–23.04	17.66	0.61	0.28	15.46	1.92	
	2019TAN (E9)	30.31	49.14	36.15	7.02	22.65–56.31	19.41	0.62	0.09	146.49	9.71	
	2019TAL(E10)	18.49	33.20	23.08	4.61	12.74–37.50	19.98	0.68	0.16	60.39	4.00	
	BLUP	26.12	40.76	31.79	0.39	23.57–42.71	1.23	0.39	-0.25	210.25	14.12	

SD, standard deviation; *CV* (%), coefficient of variation; *H*^*2*^, broad-sense heritability

FLL, flag leaf length (cm); FLW, flag leaf width (cm); FLA, flag leaf area (cm^2^). BLUP, best linear unbiased prediction. TAN had a normal phosphorus concentration in 2015–2016 (E1), 2016–2017 (E3), 2017–2018 (E6), and 2018–2019 (E9); TAL had a low phosphorus concentration in 2015–2016 (E2), 2016–2017 (E4), 2017–2018 (E7), and 2018–2019 (E10); and ZB had drought in 2016–2017 (E5) and 2017–2018 (E8)

### QTL detection of flag leaf-related traits

The mapping population consisted of 296 RILs derived from SN304 and YN15. Polymorphic markers were developed for genetic map construction using specific locus amplified fragment sequencing (SLAF-seq) technology. The genetic map included 18 groups and 3,053 loci spanning 1401.44 cM with an average genetic distance of one marker per 0.46 cM. Due to the similar genetic background of the parents, there were multiple large deletions on the RIL population genetic linkage map, and molecular markers for chromosomes 1 A, 2 A, and 5 A were not obtained. This genetic map was used to filter QTL in the present study (Additional file 2).

**Fig. 1:**
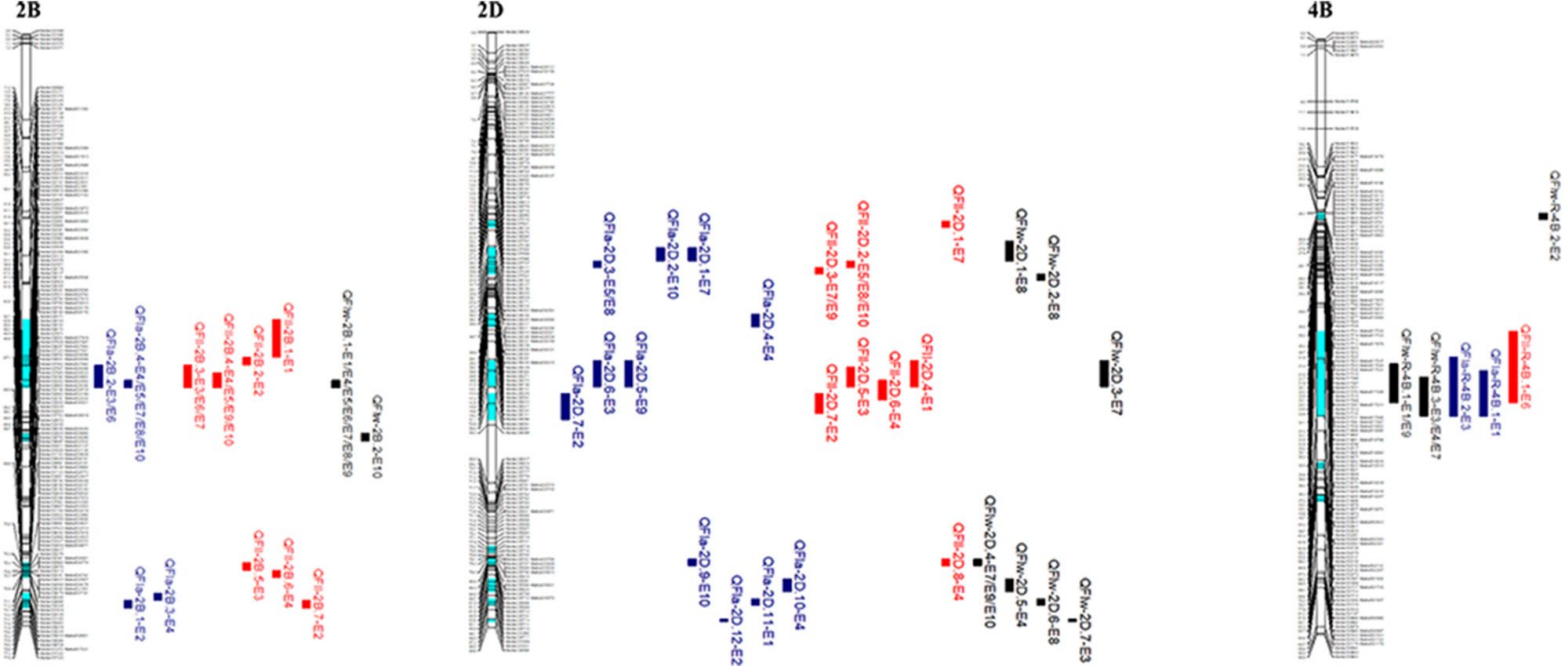
Distribution of major QTLs identified on chromosomes 2B, 2D, and 4B in ten different environments. *Note* FLL, flag leaf length; FLW, flag leaf width; FLA, flag leaf area. Map distances (cM) are indicated on the leaf of each chromosome, and marker names are on the right. A red rectangle indicates a QTL associated with FLL, a black rectangle indicates a QTL associated with FLW, and a blue rectangle indicates a QTL associated with FLA. Genetic linkage maps were constructed using the software JoinMap 4.1 and MapChart 2.3

In the ten environments and the BLUP dataset, we detected 77 QTLs for FLL, FLW, and FLA on 16 chromosomes, excluding 1 A, 2 A, 5 A, 6 A, and 7B. Among these, 14 major QTLs were found, with three QTLs identified in more than four different environments, and 19 QTLs were detected in fewer than two different environments. These QTLs individually explained 1.52-22.82% of the phenotypic variance, with LOD values ranging from 3.01 to 42.54 in different environments. The positive alleles of 51 QTLs were contributed by SN304, and the remaining 26 had positive alleles from YN15 (Additional file 5; Fig. 1).

For FLL, we identified 28 QTLs in eleven environments, 21 of which contained positive alleles from SN304 that improved the flag leaf length (Additional file 5). We found four major QTLs on chromosomes 2B and 2D. *QFll-2B.4* was identified as a major and stable QTL in four environments, mapping to the interval between markers 522,975 and 522,687 and explaining 11.27-19.24% of the phenotypic variance. *QFll-2D.5* and *QFll-2D.6* were detected in a single environment, explaining 14.61% and 10.38% of the phenotypic variation, respectively, with the positive alleles from SN304 (Additional file 5; Fig. 1).

For FLW, we detected 22 QTLs on chromosomes 1B, 2B, 2D, 3D, 4 A, 4B, and 6D, accounting for 2.48-12.97% of the phenotypic variation. Among them, 10 QTLs carried positive alleles from SN304, which increased the FLW (Additional file 5, Fig. 1). On chromosomes 2B (three QTLs) and 4B (three QTLs), we found six significant QTLs. One major QTL, *QFlw-2B.1*, was stably detected in seven environments, explaining 5.43-12.97% of the phenotypic variation, and was mapped to the interval between markers 522,975 and 522,687. Another major QTL, *QFlw-2B.2*, was found in one environment, explaining 12.81% of the phenotypic variation; *QFlw-4B.3* was found in two environments, explaining 5.99-10.8% of the phenotypic variance; and *QFlw-4B.4* was found in three environments, explaining 7.22-10.30% of the phenotypic variance. *QFlw-2B.3* and *QFlw-4B.5* were detected in BLUP dataset.

In FLA, we detected a total of 27 QTLs in different environments and BLUP dataset, explaining 1.52-22.82% of the observed phenotypic variation. These QTLs were located on chromosomes 1B, 2B, 2D, 3B, 3D, 4 A, 4B, 4D, 5D, and 7 A (Additional file 5, Fig. 1). Among them, 17 QTLs had positive alleles derived from SN304, which is known to enhance FLA. One major and stably expressed QTL, *QFla-2B.4*, was detected in five environments and the BLUP dataset, explaining 3.48-19.81% of the phenotypic variance, and was mapped to the same marker interval as FLL and FLW. Another major QTL, *QFla-2B.2*, was detected in two environments, explaining 9.10-15.28% of the phenotypic variance. While *QFla-3B* explained 22.82% of the phenotypic variance, it was detected in a single environment. *QFla-4D.2* was detected at BLUP dataset, explaining10.25% of the phenotypic variance.

In addition, we detected a total of 28 QTLs for flag leaf-related traits in a low-phosphorus environment (E2, E4, E7 and E10), explaining 1.52-22.82% of the phenotypic variance. Among them, *QFla-3B*, *QFlw-2B.2* and *QFll-2D.6* explained more than 10% of the phenotypic variance and carried the positive alleles from SN304 that increased flag leaf size. In a drought environment, we detected eight QTLs on chromosomes 4 A, 4B, 6B, 2D, and 3D, explaining 3.48-8.95% of the phenotypic variance. These QTLs, which are specifically expressed in low-phosphorus and drought environments, will play an important role in the breeding of wheat plants that are tolerant to abiotic stress.

We found twelve colocalized regions for flag leaf-related traits (Table 2), with four intervals detected for FLL, FLW, and FLA on chromosomes 2B and 2D. These intervals included positive alleles derived from the SN304 locus. The QTLs for FLL (*QFll-2D.7* and *QFll-2D.5*) FLA (*QFla-2D.7* and *QFla-2D.6*) were found to be colocalized on chromosome 2D. Similarly, the QTLs on chromosome 2B for FLL (*QFll-2B.4* and *QFll-2B.3*), FLW (*QFlw-2B.1*), and FLA (*QFla-2B.4* and *QFla-2B.2*) were also colocalized. These QTLs were mapped to the interval between markers 522,975 and 522,687 in multiple environments, suggesting that this interval could control the FLL, FLW, and FLA simultaneously. The SN304 allele of the colocated QTL was significantly associated with greater leaf size (Additional file 6).

**Table 2:** Regions for the QTL clustering of flag leaf-related traits

Codes	Traits	QTL	Chr.	Env.	LOD value	Markers interval	PVE	Add
1	FLL	*QFll-2B.4*	2B	E4/E5/E9/E10	11.36/10.58/15.10/13.24	Marker522975-Marker522687	11.27/13.85/19.24/17.04	-0.75/-0.64/-1.08/-0.87
	FLW	*QFlw-2B.1*	2B	E1/E4/E5/ E6/E7/E8/E9	12.80/6.98/4.32/8.06/7.76/5.92/11.62		10.93/5.58/5.43/11.79/8.31/6.69/12.97	-0.07/-0.05/-0.05/-0.07/-0.05/-0.03/-0.07
	FLA	*QFla-2B.4*	2B	E4/E5/E7/E9/E10	8.05/9.60/4/8.43/11.19/17.26		3.48/12.48/10.46/18.34/19.81	-1.61/-1.49/-1.36/-2.75/-2.09
2	FLL	*QFll-2B.3*	2B	E3/E6/E7	7.88/7.71/4.91	Marker521913-Marker522975	9.44/9.28/4.20	-0.88/-0.76/-0.45
	FLA	*QFla-2B.2*	2B	E3/E6	6.86/12.12		9.10/15.28	-2.11/-2.23
3	FLL	*QFll-2B.7*	2B	E2	3.16	Marker535248-Marker539249	4.39	-0.60
	FLA	*QFla-2B.1*	2B	E2	4.10		5.04	-1.47
4	FLL	*QFll-2D.7*	2D	E2	6.28	Marker226084-Marker226099	8.96	-0.86
	FLA	*QFla-2D.7*	2D	E2	4.84		5.99	-1.60
5	FLL	*QFll-2D.5*	2D	E3	11.88	Marker226098-Marker226054	14.61	-1.09
	FLA	*QFla-2D.6*	2D	E3	6.73		8.91	-2.08
6	FLL	*QFll-2D.8*	2D	E4	3.46	Marker225694-Marker225569	3.20	0.40
	FLW	*QFlw-2D.4*	2D	E7/E9/E10	5.25/3.46/5.40		5.42/3.58/6.93	0.04/0.04/0.04
	FLA	*QFla-2D.9*	2D	E10	4.48		4.61	1.01
	FLA	*QFla-2D.11*	2D	E1	6.73		8.83	-0.99
7	FLW	*QFlw-2D.3*	2D	E7	3.04	Marker226121-Marker226098	3.19	-0.03
	FLA	*QFla-2D.5*	2D	E9	4.78		7.53	-1.76
8	FLL	*QFll-2D.2*	2D	E5/E8/E10	5.34/7.57/31.51	Marker226127-Marker226126	6.62/8.01/9.34	-0.44/-1.07/-0.64
	FLA	*QFla-2D.3*	2D	E5/E8	3.32/5.63		3.99/8.95	-0.84/-0.74
9	FLW	*QFlw-2D.1*	2D	E8	5.74	Marker227524-Marker226133	6.86	-0.03
	FLA	*QFla-2D.1*	2D	E7	5.11		6.13	-1.04
	FLA	*QFla-2D.2*	2D	E10	6.74		7.05	-1.25
10	FLW	*QFlw-2D.7*	2D	E3	3.18	Marker231831-Marker230990	3.25	0.04
	FLA	*QFla-2D.12*	2D	E2	3.27		4.05	1.32
11	FLW	*QFlw-3D.1*	3D	E1/E4	4.82/4.86	Marker185080-Marker184951	3.86/3.80	-0.04/-0.04
	FLA	*QFla-3D*	3D	E1	5.05		4.37	-1.56
12	FLL	*QFll-4B*	4B	E6	3.48	Marker509207-Marker502840	4.00	-0.50
	FLW	*QFlw-4B.2*	4B	E5	4.15		5.25	-0.05
	FLW	*QFlw-4B.3*	4B	E1/E9	11.27/5.18		10.86/5.99	1.40
	FLW	*QFlw-4B.4*	4B	E3/E4/E7	8.94/9.39/6.08		10.30/9.04/7.22	-26.67
	FLA	*QFla-4B.1*	4B	E3	4.13		6.24	-1.76
	FLA	*QFla-4B.2*	4B	E1	4.55		4.74	-1.62

*Note* Chr.: chromosome, Env.: environment, PVE: phenotypic variance explained, LOD: logarithm of odds, Add: additive effect

### Identifying the relationships between major QTLs and *Th. intermedium*

**Fig. 2:**
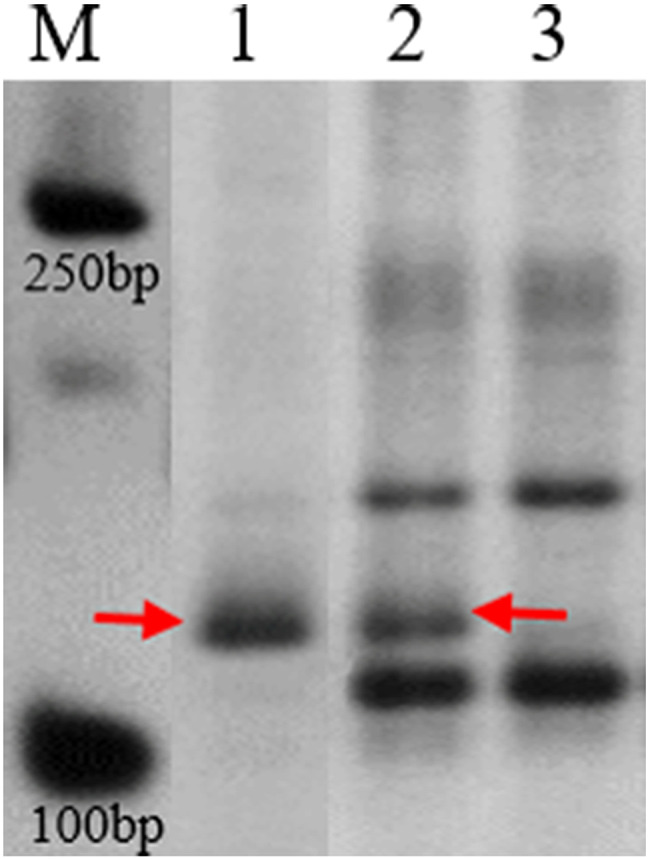
Polyacrylamide gel plots of double-ended anchored primers from the QTL interval between markers 522,975 and 522,687. *Note* The forward sequence and reverse sequence of the double-ended anchoring primers were GGCACCCGGACATCAGTT and GGGGCTAAGACAAGTCTACCAG, respectively. The red arrows indicate DNA fragments specific to *Th. intermedium*. M, marker (2 kb ladder); 1, *Th. intermedium*; 2, SN304; 3, YN15. The groups of the gel were cropped from different parts of the same gel, and the original gel is shown in Additional file 3

To investigate the potential relationship between the introgression of *Th. intermedium* chromosomes and the QTLs associated with flag leaf traits, we designed a total of 127 double-ended anchor primers. These primers were based on the sequences of the major QTLs, specifically targeting the positive alleles from SN304. We also conducted molecular marker analysis on SN304, YN15, and *Th. intermedium*. The results showed that only one pair of primers mapped to chromosome 2B produced a specific band corresponding to *Th. intermedium* in SN304 (Fig. 2).

In conclusion, we speculated that the main QTL interval on chromosome 2B affecting flag leaf-related traits was derived from *Th. intermedium*.

### Potential candidate genes for *QFll-2B.4/QFlw-2B.1/QFla-2B.4*

According to the CS reference genome (IWGSC RefSeq v2.1), there were 34 annotated high-confidence genes in the same interval of *QFll-2B.4*/*QFlw-2B.1*/*QFla-2B.4* (Additional file 5). Expression pattern analysis revealed that 11 genes were expressed in the leaves (TPM > 2) (Additional file 4). Gene annotation and orthologous gene analyses (Additional file 7), combined with previous expression pattern analysis, suggested that *TraesCS2B02G096200, TraesCS2B02G096300, TraesCS2B02G097100* and *TraesCS2B02G09730* were likely to be associated with flag leaf development and growth. *TraesCS2B02G096200* plays an important role in plant growth and development by scavenging reactive oxygen species. *TraesCS2B02G096300* is required for chloroplast division before ARC5 and utilizes arogenate more efficiently than prephenate. *TraesCS2B02G097100* and *TraesCS2B02G09730* promote the GTP-dependent binding of aminoacyl-tRNA to the A-site of ribosomes during protein biosynthesis.

## Discussion

### Multiple environment-based correlation analyses of flag leaf-related traits

In this study, the broad-sense heritabilities (*H*^*2*^) of FLL, FLW, and FLA were high, reaching 92%, 94%, and 92%, respectively. The flag leaf-related traits were more influenced by genetics than by the environment, suggesting the need to identify QTLs for leaf-related traits based on multiple environments to increase reliability.

Flag leaf size influences wheat growth and development and contributes to the photosynthetic capability of wheat, making it an important component of wheat breeding programs [35]. In our study, the correlations between flag leaf-related traits (FLL, FLW, FLA) and yield-related traits (SL, SPN, KNPS) were significant and positive (Additional file 6). These results are consistent with those of previous studies [13, 27, 30–32, 34] showing that longer and larger leaves can significantly increase spikelet and grain production by increasing photosynthesis accumulation. The correlations of FLL, FLW, and FLA and HD of and FD were also significant and positive, and similar studies [24, 36] have shown that the heading date and flowering date affect the flag leaf traits of lengthening HD and FD.

### Comparison of QTL associations with flag leaf-related traits

**Fig. 3:**
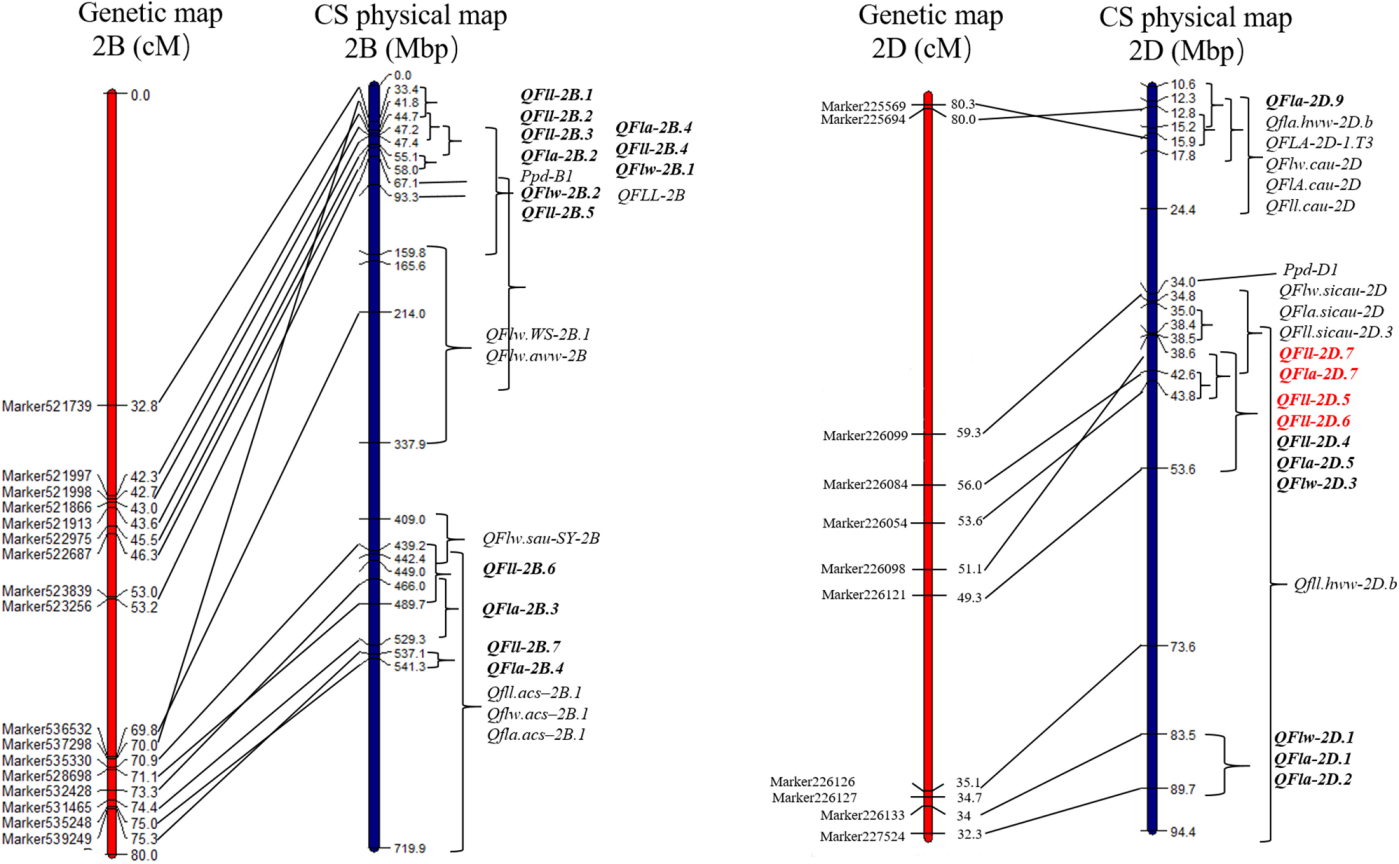
Maps of the QTLs included in this study and previous studies on chromosomes 2B and 2D. The QTLs identified in this study are shown in bold font

Here, thirteen major QTLs that colocalized and were stably expressed in multiple environments for flag leaf-related traits were identified on chromosomes 2B and 2D. To compare the intervals between the QTLs detected in our study and those previously discovered, we physically located these QTLs on the target chromosomes of CS (Fig. 3, Additional file 5). Compared mapping revealed that the genetic location of the QTLs on chromosomes 2B and 2D was consistent with the physical location in Chinese Spring.

The major QTLs located in the same interval of 2.0 cM were located between 55.1 Mbp and 58.0 Mbp on chromosome 2BS. The two intervals of *QFlw-2B.2* and *QFla-2B.2* were located at 93.26-93.27 Mb and 47.2-55.07 Mb on chromosome 2BS, respectively (Fig. 3). These intervals overlapped with *QFLL-2B*, which is located at a large distance between 47.2 Mb and 165.57 Mb [30]. These findings indicated that *QFll-2B.4*, *QFll-2B.1* and *QFla-2B.4* may be the same or linked to *QFLL-2B* [30], perhaps representing new QTLs for flag leaf-related traits; however, subsequent validation experiments are needed. In addition, compared with the photoperiod gene on 2BS (*Ppd-B1*), the major QTLs were adjacent to *Ppd-B1* [37]. Furthermore, two minor QTLs, *QFla-2B.4* and *QFll-2B.7*, were located on the same interval of 537.1-541.3 Mbp on chromosome 2BL, overlapping with three reported QTLs [38]. *QFla-2B.3* and *QFll-2B.6* were also located on chromosome 2BL, partially overlapping with the four reported QTLs [32, 38]. *QFll-2B.1, QFll-2B.2, QFll-2B.3*, and *QFll-2B.5* were located on chromosome 2BS, and three of the QTLs partially overlapped with the five reported QTLs [30, 31]. Other reported QTLs related to flag leaf-related traits were mostly located on chromosome 2BL [20, 31, 32, 34, 38, 39] and had no relationship with the QTL in this study.

The four major QTLs were detected on chromosome 2D (Fig. 3). *QFll-2D.5* and *QFll-2D.6*, both within 3.0 cM, were located between 38.6 Mbp and 43.8 Mbp and between 42.6 Mbp and 43.8 Mbp, respectively. In the same interval of 4.0 cM, *QFll-2D.7* and *QFla-2D.7* were located between 34.8 Mbp and 42.6 Mbp on chromosome arm 2DS. This interval partially overlapped with a colocated QTL located between 35.0 Mbp and 38.5 Mbp [13] and was contained in the interval from *Qfll.hww-2D.b* for FLW [40]. Additionally, the colocated intervals for *QFll-2D.5, QFll-2D.6, QFll-2D.7* and *QFla-2D.7* were not linked to *Ppd-D1*, indicating that FLL, FLW, and FLA may not be correlated with *Ppd-D1*. These results suggest that the four major QTLs could be the same as the reported QTL or could be a new QTL, which needs subsequent validation.

### The different flag leaf sizes caused by the presence of *Th. intermedium*

Genetic resources have gradually become narrower with wheat domestication and decades of breeding. The exploitation of excellent genes from wild relatives into wheat can increase genetic diversity and provide new genetic resources for wheat breeding [41, 42]. For example, wild wheat relatives have been widely used in wheat breeding as a source of disease resistance [4, 5]. *Th. intermedium* is a close wild relative of wheat and has proven to be a valuable source of disease resistance genes. In our study, SN304 was identified as a wheat-*Th. intermedium* germplasm, and it has introgressed small fragments on chromosomes 2 A, 7 A, 2B, 6B, and 7B [2]. In addition, forty-nine QTLs with positive alleles from SN304 were detected on chromosomes 2B, 2D, 3 A, 3B, 3D, 4B, and 6D. Therefore, the two ends of chromosome 2B were densely labeled, which may be due to the nonexchange of an introgressive fragment of SN304, resulting in differences in the sequences of YN15 and SN304. These results suggest that the QTLs for flag leaf-related traits detected on chromosome 2B in SN304 may be from *Th. intermedium.*

## Conclusion

In summary, a total of 77 QTLs were detected for FLL, FLW, and FLA, and 19 QTLs were consistently identified on chromosomes 2B, 3B, 4B, and 2D in multiple environments and BLUP dataset. Among them, the positive alleles of 51 QTLs were contributed by SN304, and individual QTLs explained 1.52-22.82% of the phenotypic variation. Furthermore, there was a major colocated QTL on chromosome 2B, *QFll/Flw/Fla-2B*, which was delimited to a physical interval of approximately 2.9 Mb and contained 20 candidate genes. Molecular marker analysis of QTL double-ended anchor primers revealed a specific band corresponding to *Th. intermedium* in SN304 on chromosome 2B. These results support the fine mapping of the pleiotropic effects of *QFll/Flw/Fla-2B* and provide valuable molecular markers and a theoretical basis for the application of *Th. intermedium* in wheat breeding.

## Methods

### Plant material and field trials

The QTL mapping population comprised 296 RILs derived from a cross between the wheat cultivar Yannong 15 (YN15) and wheat-*Th. intermedium* introgression line Shannong 304 (SN304). YN15, the male parent, was released by the Yantai Academy of Agricultural Science, Shandong; the female parent SN304 was developed by the Tai’an Subcenter of the National Wheat Improvement Center. After the initial cross in 2010, the lines were advanced until the F_10_ generation using single-seed descent [43]. Compared to YN15, SN304 has a larger flag leaf and better yield traits. The parent line and RILs were planted in Tai’an (117.13° E, 36.18° N) and Zibo (118.05° E, 36.78° N) in different environments: TAN had normal phosphorus (72.03 mg⋅kg^− 1^) in 2015-2016 (E1), 2016-2017 (E3), 2017-2018 (E6), and 2018-2019 (E9); TAL had low phosphorus (22.96 mg⋅kg^− 1^) in 2015–2016 (E2), 2016-2017 (E4), 2017-2018 (E7), and 2018-2019 (E10); and ZBD was a drought land in 2016-2017 (E5) and 2017-2018 (E8). A randomized complete block design with three replications was used in each environment, with a 1.5 m row length, 0.25 m row spacing, and each RILs line was seeded in four rows with 30 seeds per row. All field trials were managed using standard local practices.

### Phenotype assessment

**Fig. 4:**
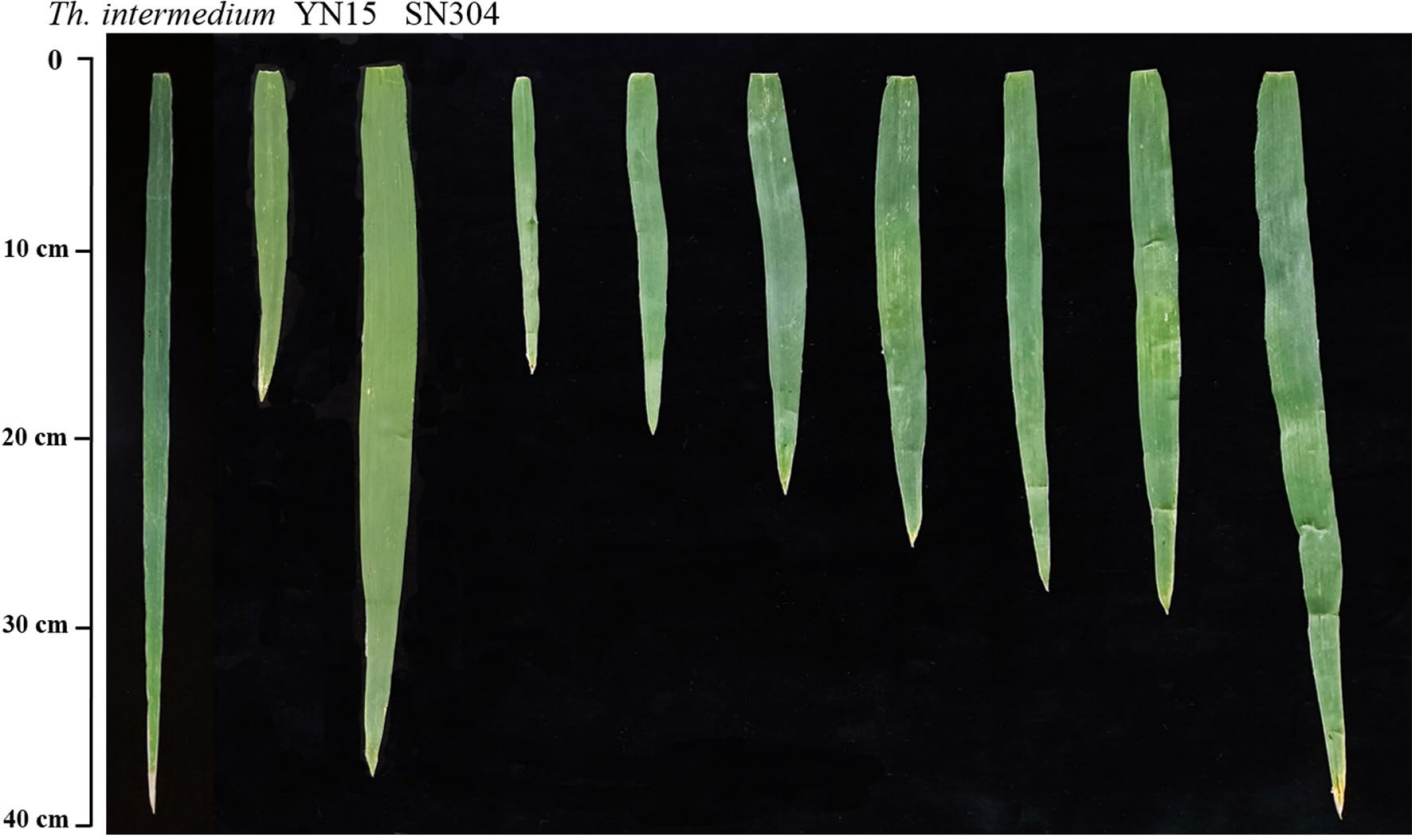
Flag leaf morphology of *Th. intermedium*, YN15, SN304, and the partial strains of the RILs population

At least eight representative plants from each line were selected to measure the FLL, FLW, and FLA after anthesis for 15 days. Flag leaves were sampled completely, photographed, and measured using an LA-S leaf area analyzer (Hangzhou Wanshen Co. Ltd.), with adjustments as necessary (Fig. 4). The methods used to measure other traits, including spikelet number per spike (SPN), grain length (KL), grain width (KW), and thousand kernel weight (TKW), were consistent with the results of previous studies [44, 45].

### Data analysis

The average value of eight typical plants per row for each line was used for subsequent analysis. A combined analysis of variance (ANOVA) was performed using the AOV function in QTL IciMapping 4.1 (http://www.isbreeding.net/). The best linear unbiased prediction (BLUP) of target traits in different environments [46].The broad heritability (*H*^*2*^ = V_G_/V_P_, where V_G_ is the genetic variance and V_P_ is the phenotypic variance) of each trait was estimated using the variance components from the ANOVA. The correlation analysis of all phenotype values was performed using SPSS version 20 software (SPSS Inc., Chicago, IL, USA; https://www.ibm.com/analytics/spss-statistics-software).

### Genetic map and QTL analysis

The Illumina HiSeq 2500 platform (Illumina, Inc., San Diego, CA, USA) was used for high-throughput sequencing. Polymorphic SLAF markers on a genome scale were screened, and SLAF-seq data were analyzed and genotyped using the method described by Sun et al. [47]. The SLAF markers were optimized, and a genetic linkage map was generated using JoinMap 4.1 with a LOD value of 10 [48]. IciMapping V4.1 software was used for individual environment QTL analysis, and the mean value for each line in each environment and the BLUP dataset were calculated [34]. The software program was run by the inclusive composite interval mapping addition (ICIM-ADD) model using the default parameter settings. The walking step was set at 1.0 cM, the PIN value was 0.001, and the LOD threshold was set at 3.0 to determine significant QTLs.

### QTL nomenclature

All QTLs are specified as follows: The capitalized italicized letter ‘*Q*’ represents ‘QTL’. The letters following the ‘*Q*’ and before the dash indicate the abbreviations of the corresponding traits. The letters and numbers following the dash represent the wheat chromosome on which the QTL were located. If several QTLs associated with a certain trait were found on a specific chromosome, the numbers (1, 2, 3, etc.) were used after the chromosome name to describe their order. When two or more overlapping QTLs related to the same trait were detected in different environments, they were considered consistent QTLs. A major QTL was defined as having an LOD > 3.0 and a phenotypic variance explained (PVE) > 10%. A major QTL was considered significant if it was detected in at least two of the ten environments. A positive additive effect indicated that the synergistic gene came from YN15, while a negative value indicated that the synergistic gene came from SN304.

### Physical intervals of major QTLs and prediction of candidate genes

The probe sequences of flanking markers for previously reported QTLs or genes related to flag leaf-related traits on chromosomes 2D, 2B, 3B, and 4B were used for BLAST against the genome assemblies of the CS reference genome IWGSC RefSeq v2.1 (http://www.wheatgenome.org) [49] to determine their physical locations. The annotations and functions of genes related to flanking markers were further analyzed using UniProt (https://www.uniprot.org/). The expression patterns of the candidate genes were analyzed using the Gene Expression of *Triticeae* Multiomics Center (http://202.194.139.32/expression/wheat.html) [50] and Expression Visualization and Integration Platform (expVIP, http://www.wheat-expression.com).

### Electronic supplementary material

Below is the link to the electronic supplementary material.

Supplementary Material 1

Supplementary Material 2

Supplementary Material 3

Supplementary Material 4

Supplementary Material 5

Supplementary Material 6

Supplementary Material 7

## Data Availability

The datasets generated and analyzed during the current study are not publicly available due to future manuscripts but are available from the corresponding author upon reasonable request. The source of the plant material used in the study was developed and preserved by our group.

## Abbreviations

CS: Chinese Spring
FD: flowering date
FLA: flag leaf area
FLL: flag leaf length
FLW: flag leaf width
HD: heading date
KNPS: kernel number per spike
LOD: logarithm of odds
PH: plant height
PVE: phenotypic variance
QTLs: quantitative trait loci
RIL: recombinant inbred line
SL: spike length
SLAF-seq: specific locus amplified fragment sequencing
SPN: spike number per plant
TKW: thousand kernel weight
