# Supplementary material for: QTL mapping for the flag leaf-related traits using RILs derived from Trititrigia germplasm line SN304 and wheat cultivar Yannong15 in multiple environments
Source: BMC Plant Biol. 2024 Apr 18;24:297. doi: 10.1186/s12870-024-04993-x (PMC11025246; doi:10.1186/s12870-024-04993-x)
Supplement: Supplementary file 1 — Supplementary Material 1 [file 12870_2024_4993_MOESM1_ESM.docx]

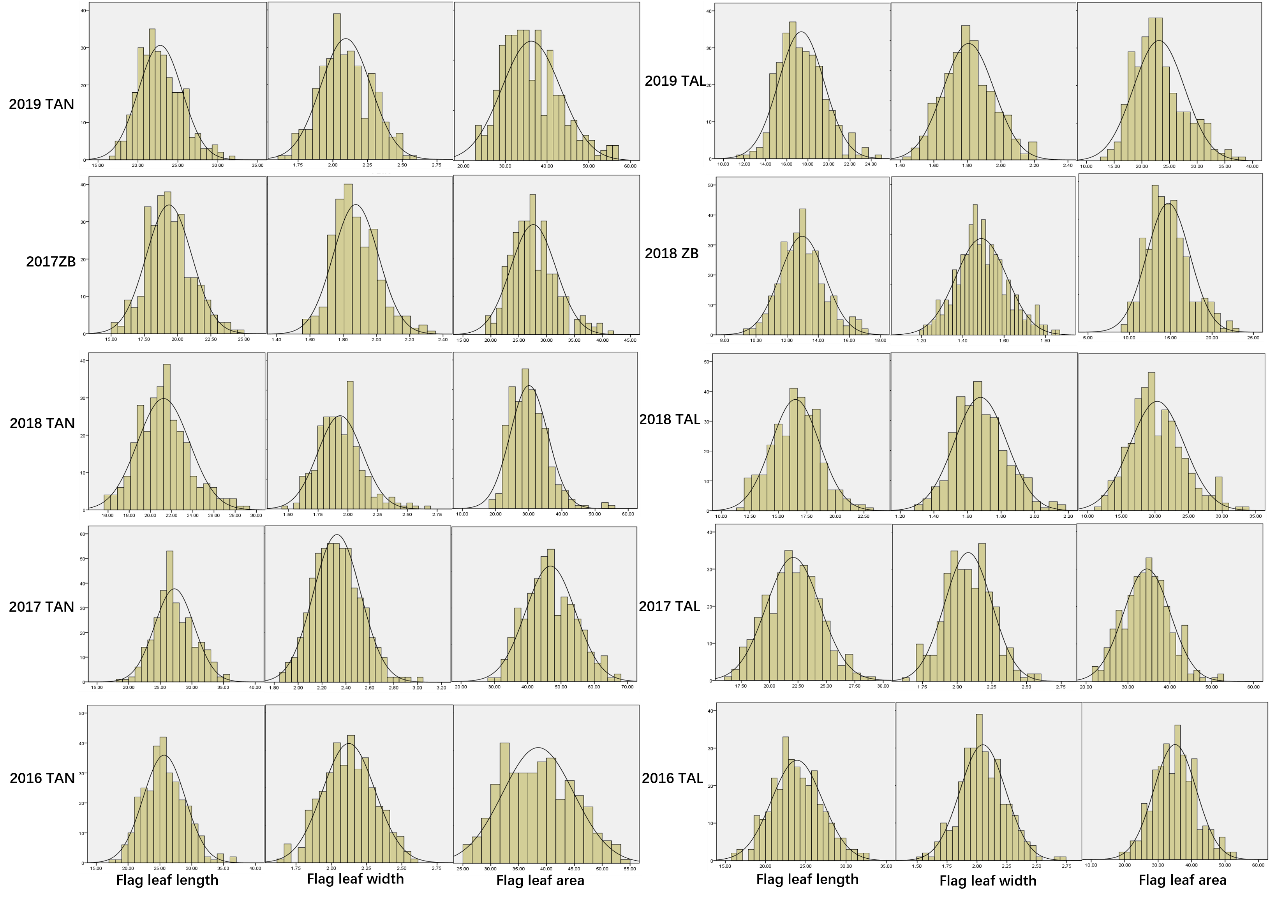


**Additional file 1.** Phenotypic distribution of wheat flag leaf size in the RILs.

The abscissa shows the mean values of flag leaf size and the ordinate represents the frequency of distribution. Letters below the graghs indicate the corresponding traits, and the markings on the left of the diagrams indicate different years and environments.
