# Supplementary material for: QTL mapping for the flag leaf-related traits using RILs derived from Trititrigia germplasm line SN304 and wheat cultivar Yannong15 in multiple environments
Source: BMC Plant Biol. 2024 Apr 18;24:297. doi: 10.1186/s12870-024-04993-x (PMC11025246; doi:10.1186/s12870-024-04993-x)
Supplement: Supplementary file 2 — Supplementary Material 2 [file 12870_2024_4993_MOESM2_ESM.docx]

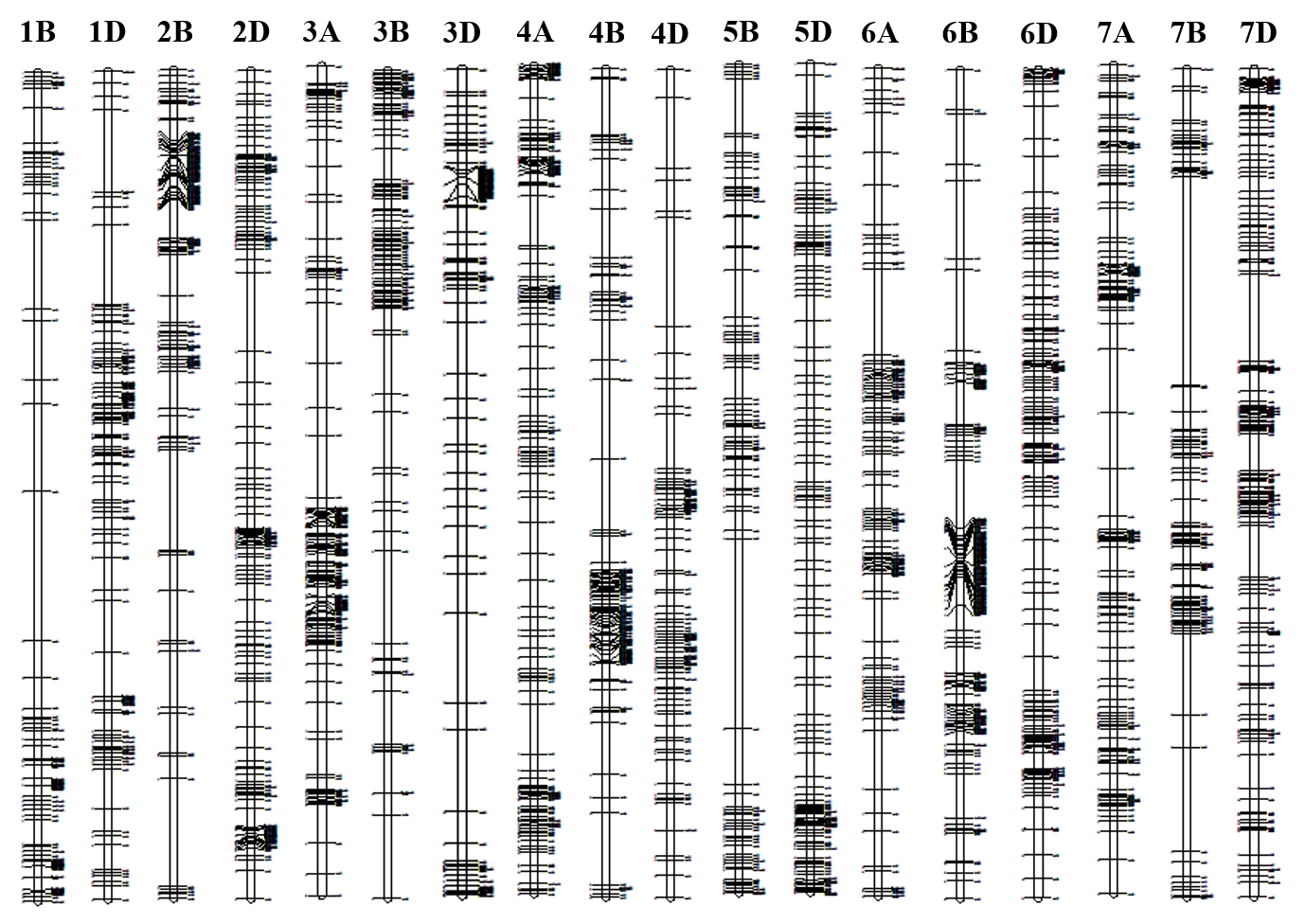


**Additional file 2.** Genetic linkage maps developed using RIL population from the cross between YN15 and SN304.
