# Supplementary material for: QTL mapping for the flag leaf-related traits using RILs derived from Trititrigia germplasm line SN304 and wheat cultivar Yannong15 in multiple environments
Source: BMC Plant Biol. 2024 Apr 18;24:297. doi: 10.1186/s12870-024-04993-x (PMC11025246; doi:10.1186/s12870-024-04993-x)
Supplement: Supplementary file 3 — Supplementary Material 3 [file 12870_2024_4993_MOESM3_ESM.pptx]

## Slide 1
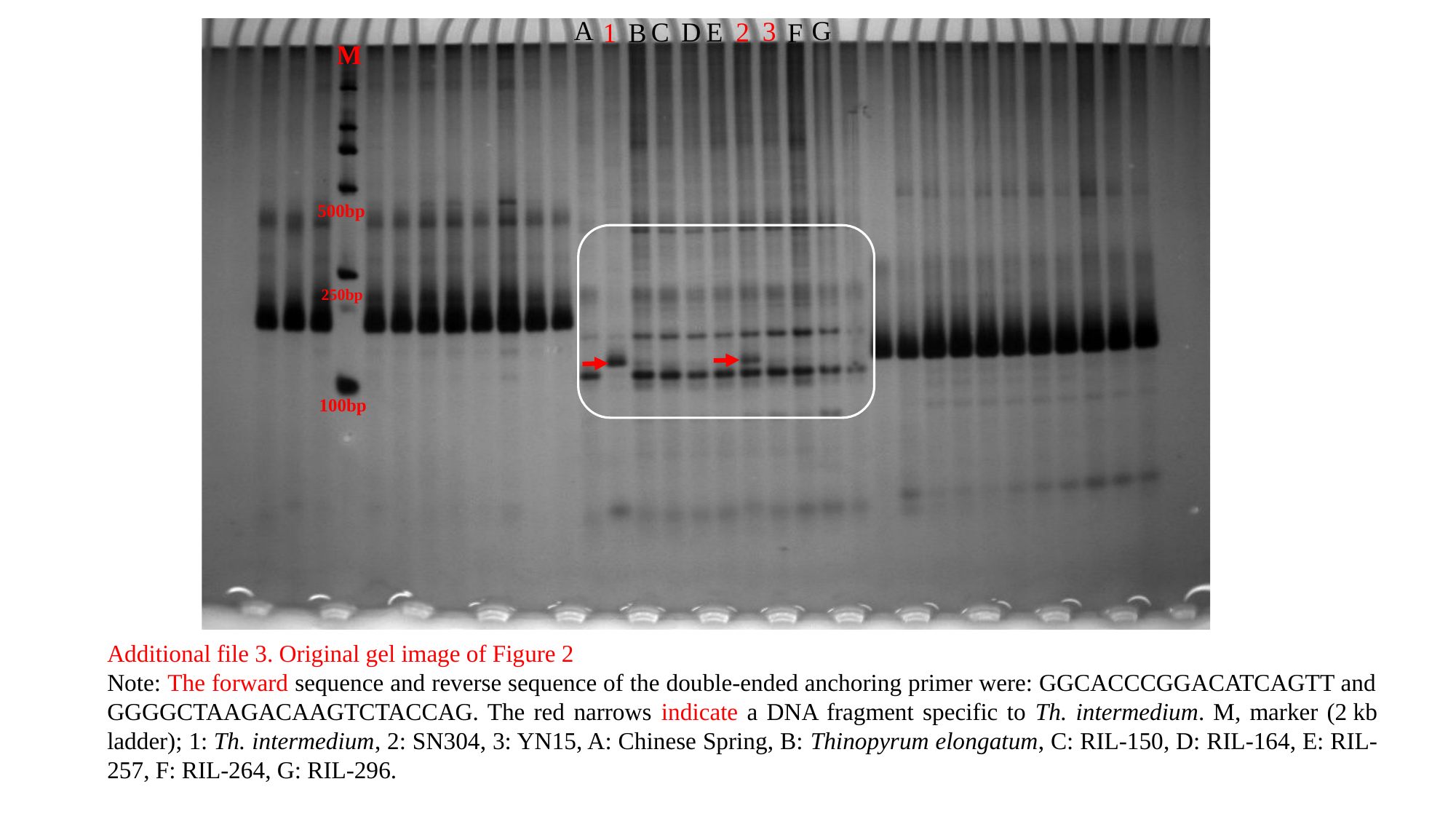

A
G
3
C
D
2
E
1
B
F
M
500bp
250bp
100bp
Additional file 3. Original gel image of Figure 2
Note: The forward sequence and reverse sequence of the double-ended anchoring primer were: GGCACCCGGACATCAGTT and GGGGCTAAGACAAGTCTACCAG. The red narrows indicate a DNA fragment specific to Th. intermedium. M, marker (2 kb ladder); 1: Th. intermedium, 2: SN304, 3: YN15, A: Chinese Spring, B: Thinopyrum elongatum, C: RIL-150, D: RIL-164, E: RIL-257, F: RIL-264, G: RIL-296.
