# Supplementary material for: QTL mapping for the flag leaf-related traits using RILs derived from Trititrigia germplasm line SN304 and wheat cultivar Yannong15 in multiple environments
Source: BMC Plant Biol. 2024 Apr 18;24:297. doi: 10.1186/s12870-024-04993-x (PMC11025246; doi:10.1186/s12870-024-04993-x)
Supplement: Supplementary file 4 — Supplementary Material 4 [file 12870_2024_4993_MOESM4_ESM.docx]

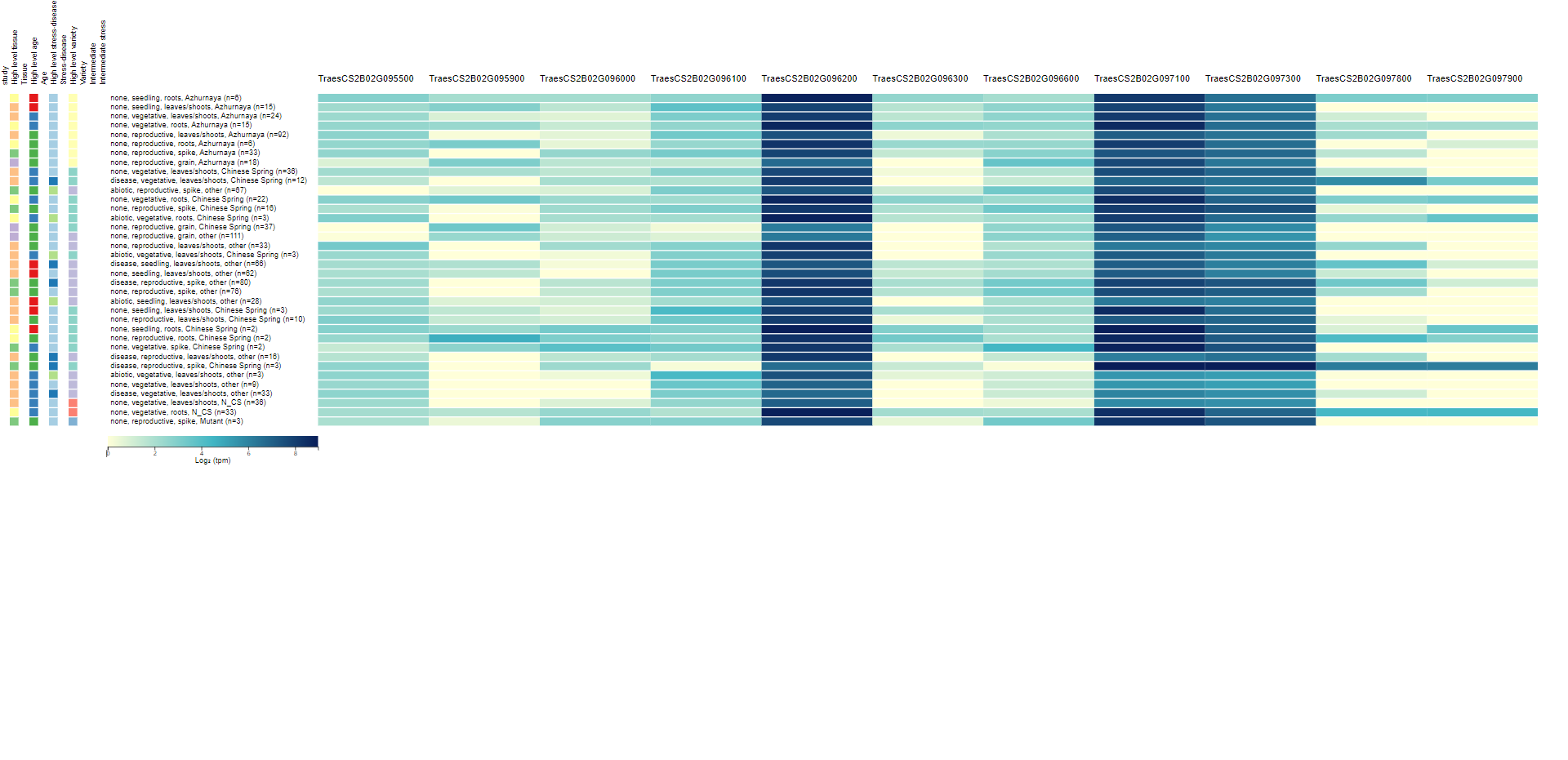


Additional file 4. Expression pattern analysis showed that 11 genes were expressed in the leaf. Note: TPM (transcripts per million) >2
