# Supplementary material for: QTL mapping for the flag leaf-related traits using RILs derived from Trititrigia germplasm line SN304 and wheat cultivar Yannong15 in multiple environments
Source: BMC Plant Biol. 2024 Apr 18;24:297. doi: 10.1186/s12870-024-04993-x (PMC11025246; doi:10.1186/s12870-024-04993-x)
Supplement: Supplementary file 5 — Supplementary Material 5 [file 12870_2024_4993_MOESM5_ESM.docx]

Additional file 5 QTL for flag leaf-related traits in all environments

| Trait | *QTL* | Env. | Left Markers | Right Markers | LOD | PVE (%) | Add |
| --- | --- | --- | --- | --- | --- | --- | --- |
| FLL | *QFll-1D* | E7 | Marker196636 | Marker201024 | 4.37 | 3.55 | 0.42 |
|  | *QFll-2B.1* | E1 | Marker521739 | Marker521997 | 5.24 | 6.91 | -0.88 |
|  | *QFll-2B.2* | E2 | Marker521998 | Marker521866 | 5.85 | 8.58 | -0.84 |
|  | *QFll-2B.3* | E3/E6/E7/BLUP | Marker521913 | Marker522975 | 4.91-13.38 | 4.20-15.75 | -0.45--0.88 |
|  | *QFll-2B.4* | E4/E5/E9/E10 | Marker522975 | Marker522687 | 10.58-15.10 | 11.27-19.24 | -0.64--1.08 |
|  | *QFll-2B.5* | E3 | Marker536532 | Marker537298 | 7.1 | 8.22 | -0.82 |
|  | *QFll-2B.6* | E4 | Marker535330 | Marker528698 | 6.55 | 6.2 | -0.56 |
|  | *QFll-2B.7* | E2 | Marker535248 | Marker539249 | 3.16 | 4.39 | -0.6 |
|  | *QFll-2D.1* | E7 | Marker227737 | Marker228141 | 10.18 | 8.59 | -0.65 |
|  | *QFll-2D.2* | E5/E8/E10/BLUP | Marker226127 | Marker226126 | 5.23-31.51 | 5,76-9.34 | -0.36--1.07 |
|  | *QFll-2D.3* | E6/E9 | Marker226333 | Marker226549 | 8.13-7.16 | 9.54-8.59 | -0.77--0.72 |
|  | *QFll-2D.4* | E1 | Marker226121 | Marker226098 | 6.73 | 8.83 | -0.99 |
|  | *QFll-2D.5* | E3 | Marker226098 | Marker226054 | 11.88 | 14.61 | -1.09 |
|  | *QFll-2D.6* | E4 | Marker226054 | Marker226084 | 10.27 | 10.38 | -0.72 |
|  | *QFll-2D.7* | E2/BLUP | Marker226084 | Marker226099 | 8.81-17.24 | 8.96-9.74 | -0.86 |
|  | *QFll-2D.8* | E4/BLUP | Marker225694 | Marker225569 | 3.46-3.53 | 3.2-3.75 | -0.47-0.29 |
|  | *QFll-3A.1* | E7 | Marker286592 | Marker286609 | 4.63 | 3.75 | -0.43 |
|  | *QFll-3A.2* | E3 | Marker286610 | Marker286761 | 4.42 | 5.02 | -0.64 |
|  | *QFll-3A.3* | E9/E10/BLUP | Marker296404 | Marker296403 | 3.20-4.98 | 3.79-5.36 | -0.35--0.51 |
|  | *QFll-3A.4* | E6/E7 | Marker296905 | Marker296761 | 3.64-5.01 | 2.92-6.04 | -0.38--0.62 |
|  | *QFll-3D.1* | E7 | Marker187500 | Marker187419 | 4.29 | 3.45 | 0.41 |
|  | *QFll-3D.2* | BLUP | Marker188466 | Marker188467 | 3.10 | 3.29 | -0.28 |
|  | *QFll-4A* | E4 | Marker355327 | Marker355328 | 3.58 | 3.35 | 0.41 |
|  | *QFll-4B* | E6 | Marker509207 | Marker502840 | 3.48 | 4 | -0.5 |
|  | *QFll-4D* | E6 | Marker179226 | Marker179154 | 3.88 | 4.41 | 0.53 |
|  | *QFll-5D* | E4 | Marker250000 | Marker250100 | 3.66 | 3.4 | 0.41 |
|  | *QFll-6B* | E5 | Marker298777 | Marker298593 | 3.24 | 4.02 | 0.34 |
|  | *QFll-7D* | E7 | Marker361599 | Marker361540 | 4.13 | 3.45 | 0.41 |
| FLW | *QFlw-1B* | E4 | Marker493273 | Marker483895 | 3.23 | 2.48 | 0.03 |
|  | *QFlw-2B.1* | E1/E4/E5/ E6/E7/E8/E9 | Marker522975 | Marker522687 | 4.32-12.80 | 5.43-12.97 | -0.03--0.07 |
|  | *QFlw-2B.2* | E10 | Marker523839 | Marker523256 | 9.59 | 12.81 | -0.05 |
|  | *QFlw-2B.3* | BLUP | Marker522975 | Marker522687 | 14.18 | 12.83 | -0.05 |
|  | *QFlw-2D.1* | E8 | Marker227524 | Marker226133 | 5.74 | 6.86 | -0.03 |
|  | *QFlw-2D.2* | E8 | Marker226171 | Marker226440 | 16.49 | 3.66 | 0.72 |
|  | *QFlw-2D.3* | E7 | Marker226121 | Marker226098 | 3.04 | 3.19 | -0.03 |
|  | *QFlw-2D.4* | E7/E9/E10 | Marker225694 | Marker225569 | 3.46-5.40 | 3.58-6.93 | 0.04 |
|  | *QFlw-2D.5* | E4 | Marker225724 | Marker225713 | 6.02 | 4.88 | 0.04 |
|  | *QFlw-2D.6* | E8 | Marker225715 | Marker231860 | 3.14 | 3.48 | 0.02 |
|  | *QFlw-2D.7* | E3 | Marker231831 | Marker230990 | 3.18 | 3.25 | 0.04 |
|  | *QFlw-2D.8* | BLUP | Marker225694 | Marker225569 | 3.90 | 3.20 | 0.03 |
|  | *QFlw-3D.1* | E1/E4 | Marker185080 | Marker184951 | 4.82-4.86 | 3.80-3.86 | -0.04 |
|  | *QFlw-3D.2* | E8 | Marker184819 | Marker184800 | 3.40 | 3.73 | -0.02 |
|  | *QFlw-4A.1* | E8 | Marker359776 | Marker359840 | 3.97 | 4.45 | 0.03 |
|  | *QFlw-4A.2* | E1 | Marker342695 | Marker342694 | 4.72 | 3.82 | 0.04 |
|  | *QFlw-4B.1* | E2 | Marker518531 | Marker518495 | 3.99 | 4.31 | -0.04 |
|  | *QFlw-4B.2* | E5 | Marker509207 | Marker502840 | 4.15 | 5.25 | -0.05 |
|  | *QFlw-4B.3* | E1/E9 | Marker509207 | Marker502840 | 5.18-11.27 | 5.99-10.86 | -0.05--0.07 |
|  | *QFlw-4B.4* | E3/E4/E7 | Marker509207 | Marker502840 | 6.08-9.39 | 7.22-10.30 | -0.05--0.08 |
|  | *QFlw-4B.5* | BLUP | Marker509207 | Marker502840 | 11.09 | 10.96 | -0.05 |
|  | *QFlw-6D* | E4/BLUP | Marker273278 | Marker274154 | 3.85/3.45 | 3.03/2.86 | -0.03/-0.02 |
| FLA | *QFla-1B.1* | E4 | Marker497580 | Marker497695 | 3.6 | 1.52 | 1.06 |
|  | *QFla-1B.2* | BLUP | Marker497281 | Marker497267 | 3.88 | 2.62 | 0.69 |
|  | *QFla-2B.1* | E2 | Marker535248 | Marker539249 | 4.1 | 5.04 | -1.47 |
|  | *QFla-2B.2* | E3/E6 | Marker521913 | Marker522975 | 6.86/12.12 | 9.10/15.28 | -2.11/-2.23 |
|  | *QFla-2B.3* | E4 | Marker532428 | Marker531465 | 4.94 | 2.14 | -1.26 |
|  | *QFla-2B.4* | E4/E5/E7/E9/E10/BLUP | Marker522975 | Marker522687 | 8.05-19.15 | 3.48-19.81 | -1.36--2.75 |
|  | *QFla-2D.1* | E7 | Marker227524 | Marker226133 | 5.11 | 6.13 | -1.04 |
|  | *QFla-2D.2* | E10 | Marker227524 | Marker226133 | 6.74 | 7.05 | -1.25 |
|  | *QFla-2D.3* | E5/E8/BLUP | Marker226127 | Marker226126 | 3.32-5.63 | 2.52-8.95 | -0.68--0.84 |
|  | *QFla-2D.4* | E4 | Marker226131 | Marker226346 | 6.67 | 2.9 | -1.46 |
|  | *QFla-2D.5* | E9 | Marker226121 | Marker226098 | 4.78 | 7.53 | -1.76 |
|  | *QFla-2D.6* | E3 | Marker226098 | Marker226054 | 6.73 | 8.91 | -2.08 |
|  | *QFla-2D.7* | E2/BLUP | Marker226084 | Marker226099 | 4.84-7.88 | 5.48-5.99 | -1--1.6 |
|  | *QFla-2D.9* | E10 BLUP | Marker225694 | Marker225569 | 4.48-8.41 | 4.61-5.87 | 1.01 -1.04 |
|  | *QFla-2D.10* | E4 | Marker225713 | Marker225714 | 5.58 | 2.38 | 1.33 |
|  | *QFla-2D.11* | E1 | Marker225715 | Marker231860 | 3.14 | 2.69 | 1.21 |
|  | *QFla-2D.12* | E2 | Marker231831 | Marker230990 | 3.27 | 4.05 | 1.32 |
|  | *QFla-3B* | E4 | Marker701184 | Marker700412 | 40.61 | 22.82 | -4.12 |
|  | *QFla-3D* | E1 | Marker185080 | Marker184951 | 5.05 | 4.37 | -1.56 |
|  | *QFla-4A* | E1 | Marker336921 | Marker336920 | 3.01 | 2.68 | 1.22 |
|  | *QFla-4B.1* | E3 | Marker509207 | Marker502840 | 4.13 | 6.24 | -1.76 |
|  | *QFla-4B.2* | E1 | Marker509207 | Marker502840 | 4.55 | 4.74 | -1.62 |
|  | *QFla-4D.1* | E6 | Marker178750 | Marker178635 | 3.39 | 3.91 | 1.13 |
|  | *QFla-4D.2* | BLUP | Marker173391 | Marker173153 | 13.98 | 10.25 | -1.36 |
|  | *QFla-5B* | BLUP | Marker482895 | Marker483088 | 2.88 | 1.92 | -0.59 |
|  | *QFla-5D* | BLUP | Marker249302 | Marker249555 | 2.58 | 1.77 | 0.57 |
|  | *QFla-7A* | E1 | Marker284701 | Marker284911 | 4.04 | 3.52 | 1.39 |

Note: Env.: environment, PVE: phenotypic variance explanation, LOD: logarithm of odds , Add: additive effect.
