# Supplementary material for: QTL mapping for the flag leaf-related traits using RILs derived from Trititrigia germplasm line SN304 and wheat cultivar Yannong15 in multiple environments
Source: BMC Plant Biol. 2024 Apr 18;24:297. doi: 10.1186/s12870-024-04993-x (PMC11025246; doi:10.1186/s12870-024-04993-x)
Supplement: Supplementary file 6 — Supplementary Material 6 [file 12870_2024_4993_MOESM6_ESM.docx]

Additional file 6 Coefficients correlations between flag leaf-related traits and yield-related traits in the RILs population.

| Traits | FLL | FLW | FLA | PH | SL | SPN | KNPS | TKW | HD | FD |
| --- | --- | --- | --- | --- | --- | --- | --- | --- | --- | --- |
| FLL | 1 |  |  |  |  |  |  |  |  |  |
| FLW | 0.51^**^ | 1 |  |  |  |  |  |  |  |  |
| FLA | 0.81^**^ | 0.85^**^ | 1 |  |  |  |  |  |  |  |
| PH | - | -0.33^**^ | - | 1 |  |  |  |  |  |  |
| SL | 0.40^**^ | 0.18^*^ | 0.32^**^ | 0.13^*^ | 1 |  |  |  |  |  |
| SPN | 0.34^**^ | 0.49^**^ | 0.52^**^ | 0.12^*^ | 0.38^**^ | 1 |  |  |  |  |
| KNPS | 0.40^**^ | 0.63^**^ | 0.56^**^ | -0.48^**^ | 0.50^**^ | 0.55^**^ | 1 |  |  |  |
| TKW | -0.14^*^ | -0.22^**^ | -0.16^*^ | 0.30^**^ | -0.20^**^ | -0.19^**^ | -0.36^**^ | 1 |  |  |
| HD | 0.38^**^ | 0.51^**^ | 0.56^**^ | 0.29^**^ | 0.32^**^ | 0.69^**^ | 0.37^**^ | -0.15^*^ | 1 |  |
| FD | 0.39^**^ | 0.56^**^ | 0.58^**^ | 0.19^*^ | 0.40^**^ | 0.71^**^ | 0.48^**^ | -0.21^**^ | 0.97^**^ | 1 |

Note：FLL, flag leaf length, FLW, flag leaf width, FLA, flag leaf area, PH plant height, SL spike length, SPN spike number per plant, KNPS kernel number per spike, TKW thousand kernel weight, HD, heading date, FD, flowering date

*Significance level at P<0.05; **significance level at P<0.01
