# Supplementary material for: QTL mapping for the flag leaf-related traits using RILs derived from Trititrigia germplasm line SN304 and wheat cultivar Yannong15 in multiple environments
Source: BMC Plant Biol. 2024 Apr 18;24:297. doi: 10.1186/s12870-024-04993-x (PMC11025246; doi:10.1186/s12870-024-04993-x)
Supplement: Supplementary file 7 — Supplementary Material 7 [file 12870_2024_4993_MOESM7_ESM.docx]

Additional file 7 Genes in the interval of the major QTLs *QFll/QFlw/Fla-2B*

| Gene ID in CS (v2.1) | Gene ID in CS (v1.0) | Orthologs in rice | Annotation |
| --- | --- | --- | --- |
| *TraesCS2B03G0221400* | *TraesCS2B02G095500* | *Os07g0695400* | Protein NETWORKED 2D |
| *TraesCS2B03G0222000* | *TraesCS2B02G095900* | *Os07g0628500* | Protein IRON-RELATED TRANSCRIPTION FACTOR 3 |
| *TraesCS2B03G0222400* | *TraesCS2B02G096000* | *Os07g0695300* | Serine/threonine-protein kinase PBL27 |
| *TraesCS2B03G0223200* | *TraesCS2B02G096100* | *Os07g0694800* | Protein RETARDED ROOT GROWTH-LIKE |
| *TraesCS2B03G0223300* | *TraesCS2B02G096200* | *Os07g0694700* | L-ascorbate peroxidase 2, cytosolic |
| *TraesCS2B03G0223400* | *TraesCS2B02G096300* | *Os07g0694600* | Arogenate dehydratase/prephenate dehydratase 2, chloroplastic |
| *TraesCS2B03G0224700* | *TraesCS2B02G096700* | *Os07g0694400* | Probable glucuronosyltransferase Os03g0287800 |
| *TraesCS2B03G0226100* | *TraesCS2B02G097100* | *Os03g0178000* | Elongation factor 1-alpha |
| *TraesCS2B03G0226300* | *TraesCS2B02G097300* | *Os03g0177900* | Elongation factor 1-alpha |
| *TraesCS2B03G0227100* | *TraesCS2B02G097800* | *-* | MLO-like protein 1 |
| *TraesCS2B03G0227200* | *TraesCS2B02G097900* | *-* | MLO-like protein 1 |
| *TraesCS2B03G0221500* | *TraesCS2B02G095600* | *Os02g0658600* | Putative expansin-B14 |
| *TraesCS2B03G0221600* | *TraesCS2B02G095700* | *Os02g0658600* | Expansin-B18 |
| *TraesCS2B03G0221800* | *TraesCS2B02G095800* | *Os07g0672700* | Anthocyanidin-3-O-glucoside rhamnosyltransferase |
| *TraesCS2B03G0224400* | *TraesCS2B02G096400* | *-* | TPD1 protein homolog 1A |
| *TraesCS2B03G0224500* | *TraesCS2B02G096500* | *Os05g0588200* | Protein PARTING DANCERS homolog |
| *TraesCS2B03G0224700* | *TraesCS2B02G096700* | *Os07g0694400* | Probable glucuronosyltransferase Os03g0287800 |
| *TraesCS2B03G0225000* | *TraesCS2B02G096800* | *Os07g0694400* | Probable glucuronosyltransferase Os03g0287800 |
| *TraesCS2B03G0226600* | *TraesCS2B02G097400* | *Os04g0390500* | Probable metal-nicotianamine transporter YSL6 |
| *TraesCS2B03G0226800* | *TraesCS2B02G097600* | *Os04g0390500* | Probable metal-nicotianamine transporter YSL6 |
